# Malignant peripheral nerve sheath tumor of the cervix in an adolescent with neurofibromatosis type 1: A case report and review of literature

**DOI:** 10.1111/jog.16139

**Published:** 2024-11-05

**Authors:** Nozomi Furuzono, Shinichi Togami, Ikumi Kitazono, Takuro Nishikawa, Akihide Tanimoto, Hiroaki Kobayashi

**Affiliations:** ^1^ Department of Obstetrics and Gynecology, Faculty of Medicine Kagoshima University Kagoshima Japan; ^2^ Department of Pathology Kagoshima University Graduate School of Medical and Dental Sciences Kagoshima Japan; ^3^ Department of Pediatrics, Graduate School of Medical and Dental Sciences Kagoshima University Kagoshima Japan

**Keywords:** malignant peripheral nerve sheath tumor, neurofibromatosis type 1, uterine cervix

## Abstract

Malignant peripheral nerve sheath tumors (MPNSTs) of the cervix are rare, particularly in patients with neurofibromatosis type 1 (NF1). This report describes a cervical MPNST in an 18‐year‐old patient with no history of sexual activity, abnormal vaginal discharge, and prolonged menstruation. She had more than six café‐au‐lait spots on her body since birth and was diagnosed with NF1 at 2 years of age. Positron emission tomography‐computed tomography revealed a large pelvic mass and lung and bone metastases. Biopsy confirmed MPNST. Immunohistochemical staining showed diffuse positivity for CD10, approximately 30% positivity for cyclin D1, partial positivity for α‐SMA, desmin, and MyoD1, and negativity for myogenin, S‐100, and SOX‐10. A cancer gene panel identified several genetic abnormalities, but none were actionable mutations. Despite systemic chemotherapy, the tumor progressed rapidly, and the patient died 8 weeks post‐admission. Early diagnosis of MPNST is crucial. In patients with NF1, even mild symptoms can indicate MPNST.

## INTRODUCTION

The occurrence of non‐epithelial malignant tumors in the cervix is rare, including mesenchymal tumors such as leiomyosarcoma, rhabdomyosarcoma, and neuroendocrine tumors. Malignant peripheral nerve sheath tumors (MPNST) commonly occur in the extremities and trunk in association with neurofibromatosis type 1 (NF1), with an incidence of approximately 2% in all patients with NF1.^1^ Its occurrence in the cervix is rare. To our knowledge, no case of MPNST in the cervix associated with NF1 has been reported. Herein, we present a case of a cervical MPNST associated with NF1.

## CASE REPORT

An 18‐year‐old woman with no history of sexual activity presented to a local gynecology clinic with chief complaints of abnormal vaginal discharge and prolonged menstruation. She experienced menarche at 12 years of age, and her menstrual cycles had been regular, occurring every 28 days. Her medical history revealed the presence of more than six café‐au‐lait spots on her body since birth, leading to a diagnosis of optic pathway glioma at the age of 2 years; this was followed by craniotomy and biopsy, leading to a diagnosis of NF1. Owing to the progressive nature of the optic pathway glioma, she underwent 10 courses of combined therapy with vincristine (1.5 mg/m^2^) and carboplatin (175 mg/m^2^) starting at 3 years of age, which resulted in tumor shrinkage. She remained under follow‐up without further treatment but developed diffuse neurofibromas throughout her body at 13 years of age. Her family history included NF1 diagnoses in her mother and maternal grandfather.

**FIGURE 1:**
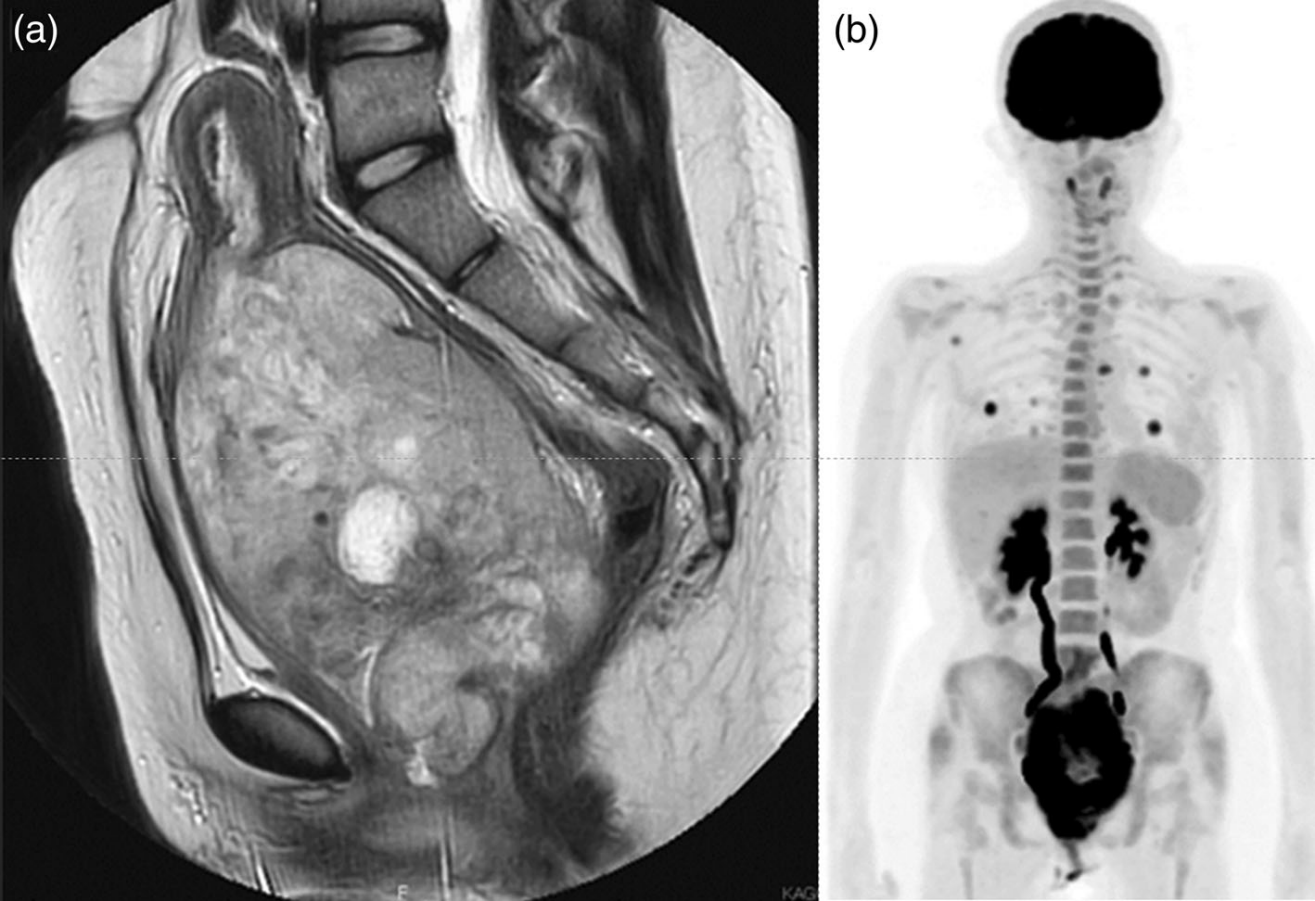
Magnetic resonance imaging with T2‐weighted images show (a) a large 14 × 10 cm tumor with high intensity in the cervix. (b) Positron emission tomography‐computed tomography revealed fluorodeoxyglucose accumulation in the cervical mass, multiple lung nodules, and the right scapula.

**FIGURE 2:**
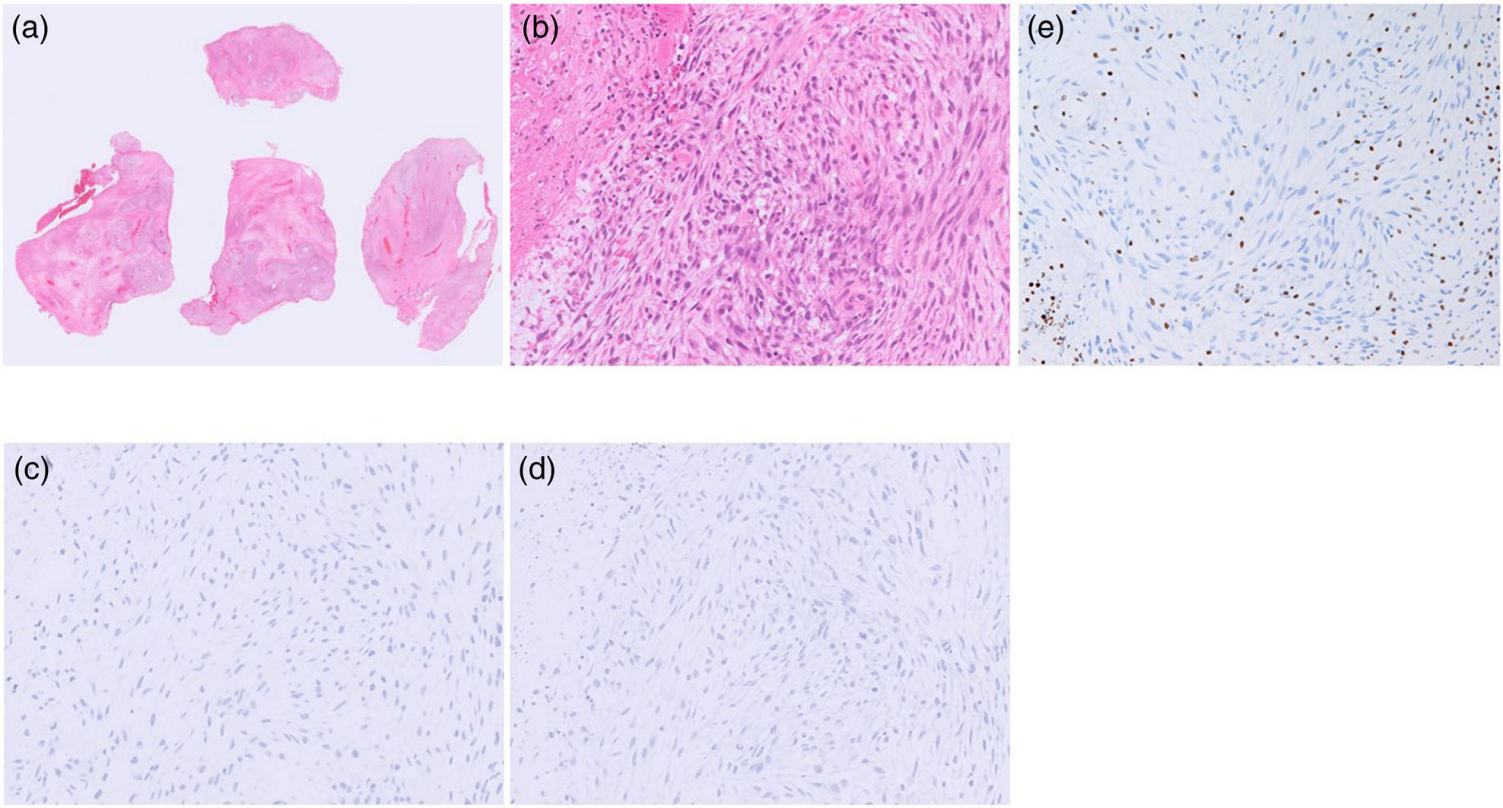
Hematoxylin and eosin staining of biopsy specimen. (a) Low‐power view reveals extensive necrosis, with tumor cells observed only in some areas around the blood vessels. (b) High‐power view reveals spindle‐shaped tumor cells proliferate in an intricate pattern. Enlarged nuclei and scattered mitotic figures are observed. Immunohistochemical staining of biopsy specimen. (c) S‐100, ×20, negative. (d) SOX10, ×20, negative. (e) Trimethylation of H3K27me3, ×20, negative.

A transrectal ultrasound revealed a solid pelvic mass the size of a fist. She was referred to our department for further evaluation and treatment, presenting with a performance status (PS) of 0. Multiple café‐au‐lait spots and neurofibromas were noted all over her body. A tumor, the size of a newborn's head, firm and elastic, was palpated in the lower abdomen. Vaginoscopy revealed a white necrotic tumor filling the vagina up to 2 cm from the vaginal introitus, adherent to the vaginal wall at the 3 o'clock position, complicating visualization of the cervix. The uterine fundus was palpable up to two transverse fingers above the umbilicus, approximately the size of a small child's head, and non‐mobile. The adnexa on both sides were not palpable. Transvaginal ultrasonography revealed a pelvic tumor with mixed echogenicity, with a maximum diameter of 13 cm, indicating a solid tumor with cystic degeneration. Blood biochemistry revealed elevated inflammatory markers with a white blood cell count of 23 150/μL and C‐reactive protein at 13.7 mg/dL, while liver and kidney functions and coagulation profiles were normal. Tumor markers included AFP at 14.6 ng/mL (normal <10 ng/mL), CA125 at 31.8 U/mL, and CA19‐9 at <2.0 U/mL. Pelvic contrast‐enhanced magnetic resonance imaging (MRI) showed a 14‐cm solid tumor, likely originating from the cervix, with low signal intensity on T1‐weighted images, high signal intensity on T2‐weighted images, and some areas of restricted diffusion. Positron emission tomography‐computed tomography revealed fluorodeoxyglucose accumulation centered on the cervix (maximum standardized uptake value [SUVmax] 13.2), multiple lung metastases (SUVmax 8.64), and metastasis to the right scapula (SUVmax 4.45) (Figure 1a,b). Enlarged lymph nodes were not observed. A biopsy of the vaginal tumor revealed spindle‐shaped to round atypical cells proliferating in bundles and an intricate pattern with necrosis. The nuclei were pleomorphic with scattered mitotic figures, suggesting a sarcoma. Additional immunohistochemical staining showed diffuse positivity for CD10, approximately 30% positivity for cyclin D1, partial positivity for α‐SMA, desmin, and MyoD1, and negativity for myogenin, S‐100, and SOX‐10 (Figure 2). Due to inconclusive histological typing from immunohistochemical staining, the biopsy tissue was submitted for further analysis in the Pediatric Solid Tumor Observation Study. Additionally, a cancer gene panel test (FoundationOne® CDx Cancer Genome Profile) was performed.

Based on these findings, a diagnosis of stage IVB cervical sarcoma was established, and chemotherapy was planned. Treatment with Adriamycin (70 mg/m^2^, every 3 weeks) was scheduled, but the patient was urgently hospitalized the day before treatment commencement due to fever and urinary retention. A bladder balloon catheter was placed, and intravenous antibiotics were administered alongside chemotherapy. On the third day of the first course, the patient developed colonic ileus due to the tumor compressing the sigmoid colon, necessitating fasting and intravenous management. On the seventh day, the patient developed febrile neutropenia, prompting treatment with granulocyte colony‐stimulating factor and intravenous antibiotics. Despite these efforts, the colonic ileus persisted, leading to the initiation of central venous nutrition by the 15th day. The patient's PS score declined from 1 to 3 during the first course of treatment. Although the neutrophil count improved by the 18th day, persistent fever raised the suspicion of tumor infection or abscess formation. Despite treatment, the tumor grew rapidly from 14 cm before treatment to 22 cm by the 19th day, as observed on contrast‐enhanced CT scan. No significant changes were observed in the metastatic lesions. Progressive disease was determined, and considering the decline in PS, we discontinued the treatment. However, at the patient and family's insistence, treatment with eribulin (1.4 mg/m^2^) was initiated. On the fifth day of the first course of treatment, the patient developed septic shock. Her overall condition gradually deteriorated, and she died on the 10th day, 9 weeks after symptom onset and 8 weeks post‐admission. An autopsy was not performed. Posthumously, the cancer gene panel test and central pathology diagnosis revealed several genetic abnormalities without actionable mutations (Table S1, Supporting Information). The central pathology diagnosis revealed complete loss of trimethylation of histone H3 on lysine 27 (H3K27me3) (Figure 2) and partial positivity for desmin without clear evidence of skeletal muscle differentiation. Considering the patient's history of NF1, she was diagnosed with a cervical MPNST. Written informed consent was obtained from the patient's parents to publish this case report, and patient anonymity was preserved.

## DISCUSSION

This report describes a cervical MPNST in an adolescent with NF1. To date, only 19 cases of cervical MPNST, including our case, have been documented in the literature.^1^, ^2^, ^3^, ^4^, ^5^, ^6^, ^7^, ^8^, ^9^, ^10^, ^11^, ^12^, ^13^, ^14^ The clinicopathological characteristics of the patients with cervical MPNST are summarized in Table 1. The typical age of onset of MPNST is in the 30s, but it occurs around adolescence in 10%–20% of cases, with an association with NF1 in 2% of cases.^1^ However, 50% of MPNST cases are associated with NF1, whereas the remaining 50% are not.^15^ MPNSTs typically occur in the buttocks, thighs, axillae, upper arms, and paraspinal regions, presenting as large soft tissue tumors that rapidly increase in size and are often accompanied by neurological symptoms or pain. Histologically, they exhibited atypical spindle‐shaped cells arranged in a fascicular pattern. Immunohistochemically, Schwann cell markers such as S‐100 protein and Sox10, are positive.^9^ However, due to the lack of distinctive histological features and specific markers, histological diagnosis can be challenging, especially in cases without NF1.

**TABLE 1:** Cases of MPNST arising in the uterus, including the present case.

No	Author	Country	Age (years)	Primary tumor	Existence of NF1	Immunohistochemistry	Tumor size (cm)
1	Sloan^13^	USA	47	Cervix	NA	S‐100+	4 × 3 × 2
2	Junge et al.^5^	NA	45	Cervix	NA	S‐100 + (focal), vimentin+, desmin‐, CK‐	1.2 × 1 × 1
3	Keel et al.^6^	USA	25	Cervix	NA	S‐100+, vimentin+, desmin‐, SMA‐, CK‐, HMB45‐	1.3
4			65	Cervix	NA	S‐100+, SMA‐, CK‐, HMB45‐	4.4 × 2.5 × 1.4
5			73	Cervix	NA	S‐100+, vimentin+, desmin‐, SMA‐, CK‐, HMB45‐	5 × 5
6	Lallas et al.^8^	USA	51	Cervix	NA	S‐100‐, vimentin+, SMA‐	3 × 3
7	Bernstein et al.^2^	USA	65	Cervix	No	S‐100+, CK‐, SMA‐	4.4 × 2.5 × 1.4
8	Di Giovannantonio et al.^3^	Italy	27	Cervix	NA	S‐100+, vimentin+, desmin‐, CK‐, HMB45‐	NA
9	Rodrigez et al.^11^	USA	22	Cervix	NA	S‐100+, CD10‐	3
10	Kim et al.^7^	South Korea	50	Cervix	NA	S‐100 + (focal), desmin‐, SMA‐, HMB45 + (focal), Melan‐A‐	6 × 3.5 × 2
11	Mills et al.^10^	USA	32	Cervix	No	S‐100 + (focal), vimentin+, CD34 + (70%), desmin‐, HMB45‐	2
12			60	Cervix	No	S‐100 + (focal), vimentin+, CD34 + (50%), desmin‐, HMB45‐	5.8
13			25	Cervix	No	S‐100 + (focal), vimentin+, CD34 + (<10%), desmin+(focal), HMB45‐	8
14	Akhavan et al.^1^	USA	53	Cervix	No	S‐100+, vimentin+, desmin+(focal), CK‐, HMB45‐	7
15	Dong et al.^4^	China	45	Cervix	NA	S‐100+	3.7 × 2.6
16	Sangiorgio et al.^12^	USA	45	Cervix	No	S‐100+, CD34 + (10%), CD10‐, desmin‐, SMA‐, HMB45‐	4
17	Zhang et al.^14^	China	46	Cervix	No	S‐100+, vimentin+, CK‐, CD34‐	NA
18	Liu^9^	China	35	Cervix	No	S‐100+, vimentin+, desmin‐, CK‐, HMB45‐, Melan‐A‐	4 × 3.5 × 2
19	Present study	Japan	18	Cervix	Yes	S‐100‐, desmin+ (focal), H3K27me3‐	14

Abbreviations: CK, cytokeratin; H3K27me3, trimethylation of histone H3 at lysine 27; HMB‐45, human melanoma black‐45; MPNST, malignant peripheral nerve sheath tumor; NA, not available; NF1, neurofibromatosis type 1; SMA, smooth muscle actin.

Previous reports have shown that 50% of MPNST cases are associated with NF1; however, this is the first case of cervical MPNST in which an association with NF1 was confirmed. The extreme rarity of cervical MPNST and lack of descriptions related to NF1 in many reports are considered reasons for this discrepancy. The loss of trimethylation of histone H3 at lysine 27 (H3K27me3) is reportedly useful for diagnosing MPNST, and a complete loss was confirmed in this case. Additionally, although the NF1 gene is located on the long arm of chromosome 17 (17q11.2), no mutations in this chromosome were detected in the cancer gene panel test. However, the absence of identified mutations in the gene panel test does not rule out NF1, as clinical diagnostic criteria must be used, and this case met the criteria for an NF1 diagnosis.

Regarding the tumor location, in this case, the cervix could not be directly observed due to the large mass occupying the vaginal cavity. However, MRI revealed that the tumor originated from and occupied the cervical region, leading to a diagnosis of cervical origin. Pathological diagnosis of MPNST in this case was supported by the presence of NF1 and confirmed by immunohistochemical staining, which revealed a loss of H3K27me3 expression. Based on these findings, we concluded that this case is an NF1‐associated cervical MPNST. In this case, the MPNST originated in the cervix following a preexisting neurofibroma. However, because the patient was a virgin and had no prior gynecological consultations, the presence of a cervical neurofibroma was unknown.

The primary treatment for MPNST is radical resection of the primary lesion, as the tumor is resistant to both radiotherapy and chemotherapy. However, early distant metastasis often occurs, making surgery unfeasible in many cases. Additionally, the recurrence rate is high, with a 5‐year survival rate of 53%, indicating a poor prognosis.^11^ MPNSTs typically have low rates of lymph node metastasis but a high incidence of lung metastasis. Prognostic factors include the size of the primary tumor and the control of lung metastasis, with primary tumors >5 cm associated with poor prognoses.^11^ In this case, the patient had poor prognostic factors, including a large‐sized primary tumor and uncontrollable lung metastases, resulting in treatment resistance.

The key insights gained from this case are as follows: First, although rare, patients with NF1 can develop MPNST in the uterus, and thus it should be considered in the differential diagnosis even with mild symptoms. Second, while MPNST tends to grow rapidly and exhibits resistance to chemotherapy, complete surgical resection may offer the potential for long‐term survival. Therefore, it is crucial to promptly perform pathological diagnosis, including genomic testing, and initiate treatment, including surgery, as soon as possible. In this case, the tumor developed in the uterine cervix, an unusual site for a disease that typically arises in the trunk, leading to a delayed diagnosis and treatment, during which the patient's PS worsened. We believe that in patients with NF1, clinicians should be vigilant for the potential development of MPNST, and prioritize rapid diagnosis and treatment, including surgery.

In conclusion, we report a case of cervical MPNST associated with NF1. Despite the administration of systemic chemotherapy, the disease could not be managed owing to the large tumor size at diagnosis and the presence of distant metastases. Although predicting MPNSTs is challenging, early diagnosis should be considered in patients with NF1, even in those with mild symptoms, such as abnormal vaginal discharge.

## CONFLICT OF INTEREST STATEMENT

Authors declare no conflict of interests for this article. Dr. Shinichi Togami is an Editorial Board member of JOGR Journal and the corresponding author of this article. To minimize bias, they were excluded from all editorial decision‐making related to the acceptance of this article for publication.

## Supporting information


**Table S1:** Results of the cancer gene panel test (FoundationOne® CDx Cancer Genome Profile).

## Data Availability

The data for this study are shown in tables and figures, and no other datasets were generated or analyzed during the current study.
