# Supplementary material for: Malignant peripheral nerve sheath tumor of the cervix in an adolescent with neurofibromatosis type 1: A case report and review of literature
Source: J Obstet Gynaecol Res. 2024 Nov 5;50(12):2372–6. doi: 10.1111/jog.16139 (PMC11608839; doi:10.1111/jog.16139)
Supplement: Supplementary file 1 — Table S1: Results of the cancer gene panel test (FoundationOne® CDx Cancer Genome Profile). [file JOG-50-2372-s001.docx]

**Supporting Information**

Supplemental Table 1: Results of the cancer gene panel test (FoundationOne® CDx Cancer Genome Profile)

| Microsatellite status | MS-stable |
| --- | --- |
| Tumor Mutational Burden | 2 Muts/Mb |
| CDKN2A | Loss |
| CDKN2B | Loss |
| EED | P161fs*5 |
| EZH2 | splice site 626-2A>G |
| MTAP | Loss |
| NF1 | Loss |
| PIK3CA | H1047R |
| PTEN | Loss |
